# Learning the hierarchical organization of the frontal lobe with differential causal effects

**DOI:** 10.1016/j.sctalk.2024.100329

**Published:** 2024-03-20

**Authors:** Kurt Butler, Duncan Cleveland, Charles B. Mikell, Sima Mofakham, Yuri B. Saalmann, Petar M. Djurić

**Affiliations:** a Department of Electrical and Computer Engineering, Stony Brook University, Stony Brook, NY 11794, USA; b Department of Neurosurgery, Stony Brook University, Stony Brook, NY 11794, USA; c Department of Psychology, University of Wisconsin-Madison, Madison, WI, USA

**Keywords:** Brain, Causal strength, Gaussian process, Medical signal processing, Network, Time series

## Abstract

In this video article, accompanying the paper “An approach to learning the hierarchical organization of the frontal lobe”, we discuss a data driven approach to learning brain connectivity. Hierarchical models of brain connectivity are useful to understand how the brain can process sensory information, make decisions, and perform other high-level tasks. Despite extensive research, understanding the structure of the prefrontal cortex (PFC) remains a crucial challenge. In this work, we propose an approach to studying brain signals and uncovering characteristics of the underlying neural circuity, based on the mathematics of Gaussian processes and causal strengths. For discovering causations, we propose a metric referred to as double-averaged differential causal effect, which is a variant of the recently proposed differential causal effect, and it can be used as a principled measure of the causal strength between time series. We applied this methodology to study local field potential data from the frontal lobe, where the interest was in finding the causal relationship between the medial and lateral PFC areas of the brain. Our results suggest that the medial PFC causally influences the lateral PFC.


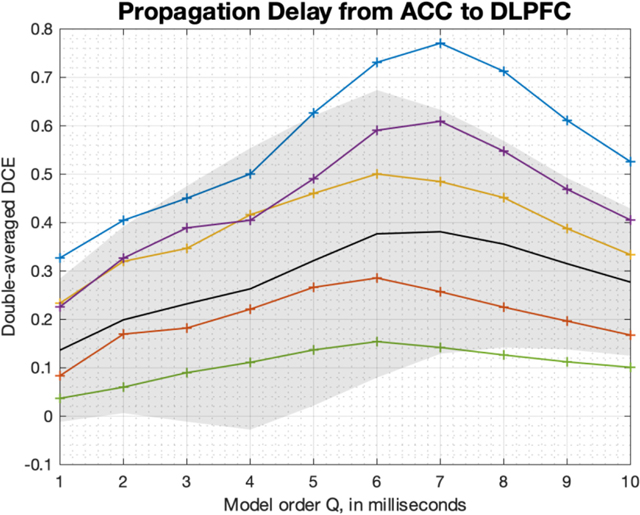


Double-averaged Differential Causal Effect (DCE) vs. the model order parameter Q. In black, we show the average across all pairs of channels and trials, with one standard deviation in the shaded region. In each pair, one channel is from the anterior cingulate cortex (ACC) and the other from dorsolateral prefrontal cortex (DLPFC). The colored curves correspond to the results for fixed pairs of channels, but we still average over trials to produce each curve. The strongest influence appears when the model order (interpreted as a window length) is around 6 to 8 ms.


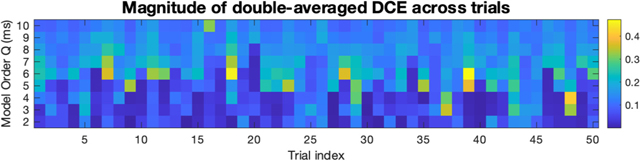


Heatmap illustrating how the double-averaged Differential Causal Effect (DCE) varies from trial to trial, for a fixed pair of channels (one from anterior cingulate cortex (ACC) and one from dorsolateral prefrontal cortex (DLPFC)). For clarity, we only show 50 trials here. There is noticeable variability from trial to trial. However, mostly all trials show the behavior that the causal strength is small for small Q, and the measured causal strength of the ACC on the DLPFC increases significantly when Q passes some trial-dependent threshold.


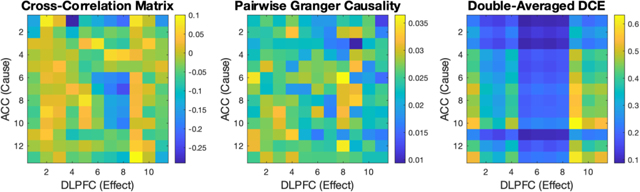


Comparison of the cross-correlation, pairwise Granger causality [1], and the double-averaged Differential Causal Effect (DCE) across pairs of channels from the anterior cingulate cortex (ACC) and dorsolateral prefrontal cortex (DLPFC), respectively. The cross-correlation and Granger causality analyses are suggestive of a relationship between ACC and DLPFC, but the resulting plots do not show recognizable spatial structure across channels. The double-averaged DCE detected causal strength which was more spatially organized than the two linear measures. In all three plots, the matrices were computed using 212 trials of the hierarchical rule task, where each channel recorded 1000 samples of data per trial.

## Figures and Tables

**Video 1:** 

## Data Availability

The authors do not have permission to share data.

## References

[1] Bressler SL, Seth AK, Wiener–Granger causality: a well established methodology, Neuroimage 58 (2) (2011) 323–329. 20202481 10.1016/j.neuroimage.2010.02.059

